# Status of work alienation among nurses in China: A systematic review

**DOI:** 10.3389/fpsyt.2022.986139

**Published:** 2022-11-08

**Authors:** Qian You, Dingxi Bai, Chenxi Wu, Jing Gao, Chaoming Hou

**Affiliations:** School of Nursing, Chengdu University of Traditional Chinese Medicine, Chengdu, China

**Keywords:** work alienation, nurse, systematic review, turnover, China

## Abstract

**Background:**

Work alienation is a common feeling of estrangement from the work and its context. Nurses are prone to feel alienated due to the high risk of infection, heavy workload, and the persistence of stress at high levels. Work alienation has serious negative outcomes, organizationally as well as personally. In recent years, the issue of work alienation among nurses has received considerable attention in China, but no systematic reviews have yet been published and its epidemiological status among Chinese nurses remains unclear.

**Objective:**

To systematically evaluate the status and distribution characteristics of work alienation among nurses in China.

**Methods:**

CINAHL, Embase, Web of Science, PubMed, CENTRAL, Wanfang, SinoMed, CNKI, and VIP were searched for cross-sectional studies before 10 January 2022 on the current status of work alienation among nurses. Two investigators independently screened the articles, extracted the data, and evaluated the risk of literature bias. Stata16.0 software was used for analysis.

**Results:**

A total of 12 studies were included, with 7,265 nurses involved. Meta-analysis results showed that the score of work alienation was 35.43 [95%*CI* (31.82, 39.04)]. Subgroup analysis showed that the scores of male and female nurses were 37.62 and 35.79 respectively; the scores of junior, undergraduate, and graduate nurses were 34.90, 37.15, and 40.02 respectively; the scores of primary, intermediate, and senior nurses were 36.95, 35.38, and 33.11 respectively; the scores of unmarried and married nurses were 38.59 and 36.70 respectively; the scores of nurses who had worked for 1~ <6 years, 6~10 years, and more than 10 years were 37.46, 36.69, and 32.89 respectively; the scores of nurses with salary <5,000 yuan, 5,000~10,000 yuan, and more than 10,000 yuan were 40.25, 37.19 and 34.52 respectively; and the scores of nurses in emergency department and intensive care units, internal medicine, surgery, and pediatrics were 37.25, 38.73, 36.28, and 31.98 respectively.

**Conclusion:**

Chinese nurses had a moderate level of work alienation. The scores of nurses in the following categories were quite high: male, higher education levels, low-professional titles, unmarried, shorter clinical working time, lower income, working in internal medicine, and working in the emergency department and intensive care units. Managers should take effective measures as soon as possible to reduce the occurrence of nurses' sense of work alienation.

**Systematic review registration:**

https://www.crd.york.ac.uk/PROSPERO/, identifier CRD42022298746.

## Introduction

Work alienation, a common phenomenon among employees, is defined as employees' feelings of estrangement from the work and its context (1). Among quick-service restaurant managers, 15.4% of them felt alienated (2). Similarly, one in five information technology professionals felt alienated (3). Work alienation has been evidenced as a negative state and related to negative repercussions such as absenteeism, anti-productivity behavior, organizational citizenship behavior, and neglect in pursuing common goals of the organization (4). It exerts a negative effect on both the organization and the individuals, disharmonizing interpersonal relationships and impeding knowledge sharing and learning (5). What's more, employees may suffer from distress and anxiety, and over time psychological problems arise.

Health sector employees feel alienated more easily, with nursing professionals being one of the most vulnerable populations to this psychological state due to the predominance of shift work, high risk of infection, heavy workloads and litigation, and high-stress levels. A previous study indicated that 87.3% of nurses experience a moderate level of workplace alienation (6). A recent study further suggested that for Chinese nurses, the status of work alienation was at a medium-high level (7). In recent years, the issue of work alienation among nurses has received considerable attention, as the growing evidence suggested that it can result in serious physical, psychological, and occupational consequences. For example, work alienation is related to low productivity, low motivation, poor performance (8), low commitment (9), low job satisfaction (10), substance use (11), and turnover intention (12). This negative state may further result in disharmonizing the nurse-patient relationship, the occurrence of missed nursing, declining patient safety and satisfaction, and finally affecting the quality of nursing care (13). Furthermore, job dissatisfaction and turnover intentions accordingly increase, which leads to the shortage of nurses, a vicious circle that work alienation has made (14). Given these findings, appropriate measures should be taken to alleviate work alienation for the sake of nursing staff, patients, and healthcare organizations.

Despite work alienation being a common feeling among nurses, healthcare managers frequently overlook the issue and its related factors, which may be attributed to the wide variation of work alienation among nurses on the one hand (7), and the lack of recognition of this psychological state on the other. Therefore, healthcare managers should improve their understanding of work alienation and its significance among nurses.

The wide variation in the status of work alienation among nurses may also be a key factor explaining the lack of attention in hospitals (7). The ignorance of work alienation may be attributed to the lack of recognition of this psychological state. By improving the understanding of the status of work alienation among nurses, healthcare managers may increase awareness of its significance.

Compared with other countries, work alienation among nurses is gaining increasing attention in China, with the body of research on work alienation among nurses growing rapidly. However, conclusions about the status of work alienation among nurses in China have been inconsistent. Some studies have reported that the status of work alienation among nurses was at a medium-high level (15), while other studies have reached different conclusions. For example, Chen et al. (16) surveyed 196 nurses in an infectious disease hospital in Nanjing, and the results showed that work alienation among nurses was at a low level. Discrepancies between studies possibly result from differences in survey time, sample size, study regions, and other factors. Moreover, no systematic review has so far been conducted on the status of work alienation among nurses in China. This review is aimed to fill the gap in our knowledge about work alienation and provide a Chinese perspective on the management of nurses around the world. Therefore, a systematical evaluation of the current status of work alienation among Chinese nurses was carried out, to provide a scientific basis for more targeted interventions to alleviate work alienation among nurses.

In essence, this study has made several important theoretical contributions. To begin with, it is the first systematic review to analyze the status of work alienation among nurses in China, which will enhance a theoretical understanding of work alienation among nurses. Second, our research is evidence-based which puts forward the concept of work alienation and treats it as a crucial aspect of the workplace that may bring negative organizational outcomes as well as personal outcomes, thus filling the gap of ignorance of work alienation (17). Third, practical recommendations are made for reference by healthcare management in our study to reduce the turnover of nursing professionals since it is tricky for managers to identify and handle work alienation, as employees usually do not share their feelings with colleagues and managers (18).

## Methods

This study aims to estimate the status of work alienation among Chinese nurses. PRISMA (Preferred Reporting Items for Systematic Reviews and Meta-Analysis) was used to report the study. The study protocol was registered under PROSPERO (Ref: CRD42022298746).

### Search strategy

Comprehensive research on PubMed, Embase, CENTRAL, Web of Science, and four Chinese databases [National Knowledge Infrastructure (CNKI), Chinese Biomedical Literatures database (SinoMed), Wanfang Digital Periodicals (WANFANG), and Chinese Science and Technology Periodicals (VIP)] were conducted to identify the status of work alienation among Chinese nurses. The following search terms and logic were used: alienation ('alienation' OR 'work alienation'). They were combined with title/abstract words related to nursing ('nurs^*^' OR 'health occupations' OR 'health personnel' OR 'health facilities' OR 'healthcare worker') through the Boolean operator AND. This search strategy was implemented in each of the databases mentioned above. There were no restrictions on the date, publication status, or year of publication, but the languages were confined to English and Chinese.

### Inclusion and exclusion criteria

The inclusion criteria were as follows: (1) cross-sectional studies; (2) Chinese nurses; (3) a scale compiled by Chinese scholar Ren was adopted, which includes twelve items and three dimensions of helplessness, powerlessness, and meaninglessness, giving a total score ranging from 12 to 60 points, in which a greater score means a high level of work alienation (19). This scale has been adopted by many scholars in China and showed good reliability and validity (20). (4) the research variable is work alienation. No limitation was exerted on gender, race, or educational status. The exclusion criteria were: (1) the studies not in English or Chinese language; (2) duplicate studies; and (3) no eligible data for extraction.

### Data extraction

Once the search was completed, the next step in data extraction was to screen the articles. Two researchers (YQ and BDX) screened the obtained literature based on the inclusion and exclusion criteria. They extracted the data, discussed it, or deliberated it with another researcher independently (WCX) when there was no agreement. The data extraction content included the research title, the first author, year of research publication, region, department, sample size, age, gender, and the total score of work alienation.

### Quality assessment

The tool of the National Center for Biotechnology Information (US) recommended by the Agency for Healthcare Research and Quality (AHRQ) was used to evaluate the quality of included studies by two reviewers (YQ and WCX) independently. Discrepancies relating to the quality of studies were resolved by a third reviewer (BDX or GJ). The AHRQ tool includes a total of 11 items with the options of “Yes (1 point)”, “No (0 points)”, and “I don't know (0 points)”. According to the score, 0–3 points are regarded as low quality, 4–7 points as medium quality, and 8–11 points as high quality (21).

### Statistical analysis

Stata 15.0 software was used for data analysis and calculating the pooled scores (using the metan command). The heterogeneity of the included studies was judged by the *Q* test (*P*-value) and *I*^2^. If *P* ≤ 0.1 or *I*^2^≥50%, it means there is statistical heterogeneity between the studies, and the random effects model is to be applied. If not, the fixed effects model would be applied for the meta-analysis. Based on gender, education, professional title, marital status, working years, salary, and department, subgroup analysis was conducted to explore the source of heterogeneity. Meanwhile, one-by-one elimination of included studies through sensitivity analysis was used to detect the stability of results and Begg's test was used to detect publication bias.

## Results

### Study search

**Figure 1:**
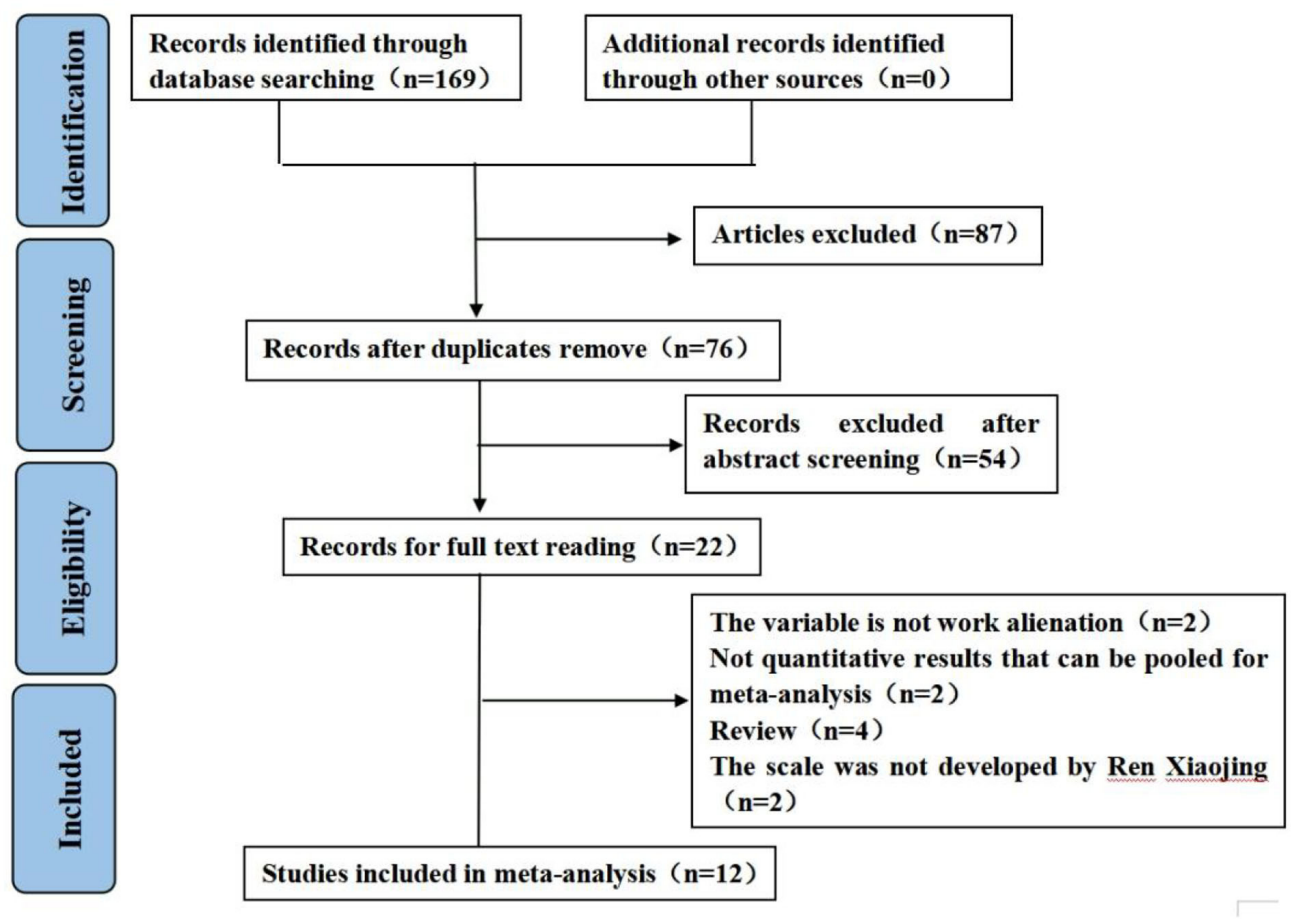
Flowchart of included studies (PRISMA-2009-Flow-Diagram). From Page et al. (22). For more information, visit: http://www.prisma-statement.org/.

A total of 169 articles were identified following our search strategy (four from PubMed, two from Embase, 20 from Web of Science, three from CENTRAL, 41 from CINAHL, 26 from CNKI, 20 from SinoMed, 32 from Wanfang, and 21 from VIP). Finally, 12 articles were included after screening (Figure 1).

### Study characteristics and risk of bias assessment results

This systematic review included 12 articles with a total of 7,265 nurses. The included articles were all of medium and high-quality–four high-quality articles and eight medium-quality articles. The basic characteristics of the included studies and the evaluation of the risk of bias are presented in Table 1.

**Table 1:** Characteristics of the included studies.

**First author (year)**	**Region**	**Department**	**Sample size**			**Age**	**Education**			**Professional** **qualifications**			**Score**	**Quality**
			**M**	**W**	**Total**		**Junior college**	**Undergraduate college**	**Postgraduate and above**	**Primary**	**Middle**	**High**		
Hu et al. (23)	He Nan	Pediatrics	7	226	233	≥20	71	146	16	215	15	3	35.02	High
Ling et al. (24)	Zhe Jiang	ICU	46	188	234	≥22	70	132	32	89	119	26	30.66	Middle
Zhang et al. (25)	Zhe Jiang	Emergency	56	231	287	-	56	209	22	166	95	26	45.49	Middle
Zhong et al. (26)	Guang Dong	Obstetrics, gynecology, emergency, operating room, internal medicine, surgery	26	577	603	≥20	232	366	5	510	84	9	35.00	High
Zhu et al. (27)	Zhe Jiang	Emergency	20	232	252	≥22	97	136	19	84	123	45	45.12	Middle
Xiao et al. (28)	Hu Bei	Internal Medicine, Surgery, Emergency, ICU	0	438	438	≥20	132	306		278	136	24	34.22	Middle
Bai et al. (29)	Zhe Jiang	Pediatrics	0	138	138	-	32	87	19	78	49	11	28.90	High
Chen et al. (20)	Zhe Jiang	Internal medicine, operating room	222	3,462	3,684	-	1,328	2,227	129	2,012	1,507	165	43.69	High
Zhao et al. (30)	Xin Jiang	Internal Medicine, Surgery, Emergency, Obstetrics, Pediatrics, ICU	19	748	767	≥20	377	390		616	116	35	30.18	Middle
Lu et al. (31)	Liao Ning	operating room	8	194	202	≥25	70	132	0	56	107	39	31.70	Middle
Xiao et al. (32)	Guang Dong	Emergency, ICU	29	316	345	-	113	230	2	239	96	10	35.91	Middle
Jiang et al. (33)	Zhe Jiang	Dialysis department	2	80	82	≥22	57	25		59	23	-		Middle

Means not reported. M/W, Men/Women; ICU, intensive care unit.

## Results of the meta-analysis

### The score of work alienation

**Figure 2:**
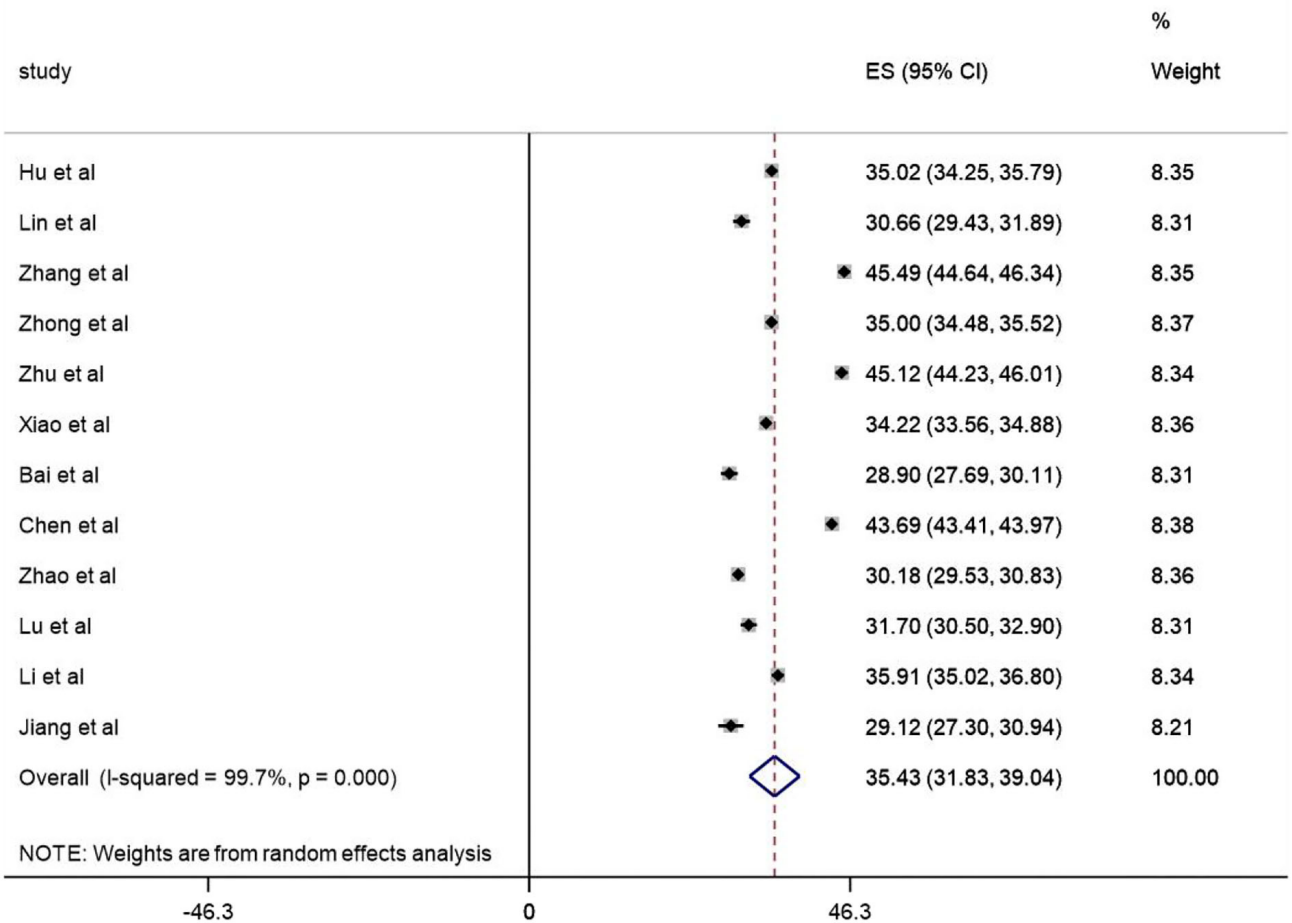
Forest plot of prevalence of PDF.

The total score of work alienation of 7, 265 nurses were extracted from the 12 finally selected articles. The quantitative evaluation results showed that the included studies had high heterogeneity (*I*^2^= 99.7%, *P* < 0.001), so a random effect model was used for meta-analysis. The total score of nurses' work alienation was 35.43 [95%*CI* (31.827, 39.040)], which was in the middle level (Figure 2). The source of heterogeneity through subgroup analysis had not been identified and a further review of the literature suggested that the generation of heterogeneity may be related to factors such as sample size, sampling method, and sampling area.

### Subgroup analysis results

Subgroup analysis was conducted on gender, education background, professional title, marital status, clinical working time, salary, and department. The results showed that the score of work alienation was quite high (Table 2).

**Table 2:** Subgroup analyses of the PDF.

**Subgroup**	**Number of studies**	**Sample size**	**Results of heterogeneity**		**Effect model**	**Results of meta-analysis**
			***I^2^*** **(%)**	***P***		**Score(95%*CI*)**
**Gender**						
Men	4	335	97.2	<0.05	Random	37.62[31.16,44.11]
Women	6	4,651	99.7	<0.05	Random	35.79[30.39,41.20]
**Education background**						
Junior college	6	1,727	98.5	<0.05	Random	34.90[30.96,38.83]
Undergraduate	5	2,837	99.4	<0.05	Random	37.15[32.31,41.99]
Postgraduate and above	4	199	96.6	<0.05	Random	40.02[34.78,45.26]
**Professional qualifications**						
Primary	6	2,679	99.1	<0.05	Random	36.95[32.80,41.11]
Middle	7	2,129	99.4	<0.05	Random	35.38[29.37,41.40]
High	7	326	97.8	<0.05	Random	33.11[26.77,39.44]
**Marital status**						
Unmarried	5	1,713	98.8	<0.05	Random	38.59[33.53,43.65]
Married	5	3,022	99.6	<0.05	Random	36.70[30.77, 42.64]
Divorced/widowed	2	110	0	>0.05	Fixed	44.14[42.86,45.44]
**Years of working**						
1~ <6	3	303	96.2	<0.05	Random	37.46[32.77,42.15]
6~10	3	318	99.1	<0.05	Random	36.69[28.22,45.17]
>10	5	423	99.1	<0.05	Random	32.89[25.03,40.74]
**Salary**						
<5,000 yuan	4	1,213	98.5	< 0.05	Random	40.25[35.45,45.06]
5,000~10,000 yuan	4	2,234	99.3	<0.05	Random	37.19[30.70,43.68]
>10,000 yuan	4	896	99.4	<0.05	Random	34.52[26.22,42.83]
**Department**						
Emergency & ICU	6	1,401	99.5	<0.05	Random	37.25[32,58, 41.91]
Internal Medicine	2	1,762	99.8	<0.05	Random	38.73[29.33,48.13]
Surgery	3	1,607	99.8	<0.05	Random	36.28[27.71,44.85]
Pediatrics	2	376	98.6	<0.05	Random	31.98[25.98,37.98]

### Sensitivity analysis/ risk of bias

**Figure 3:**
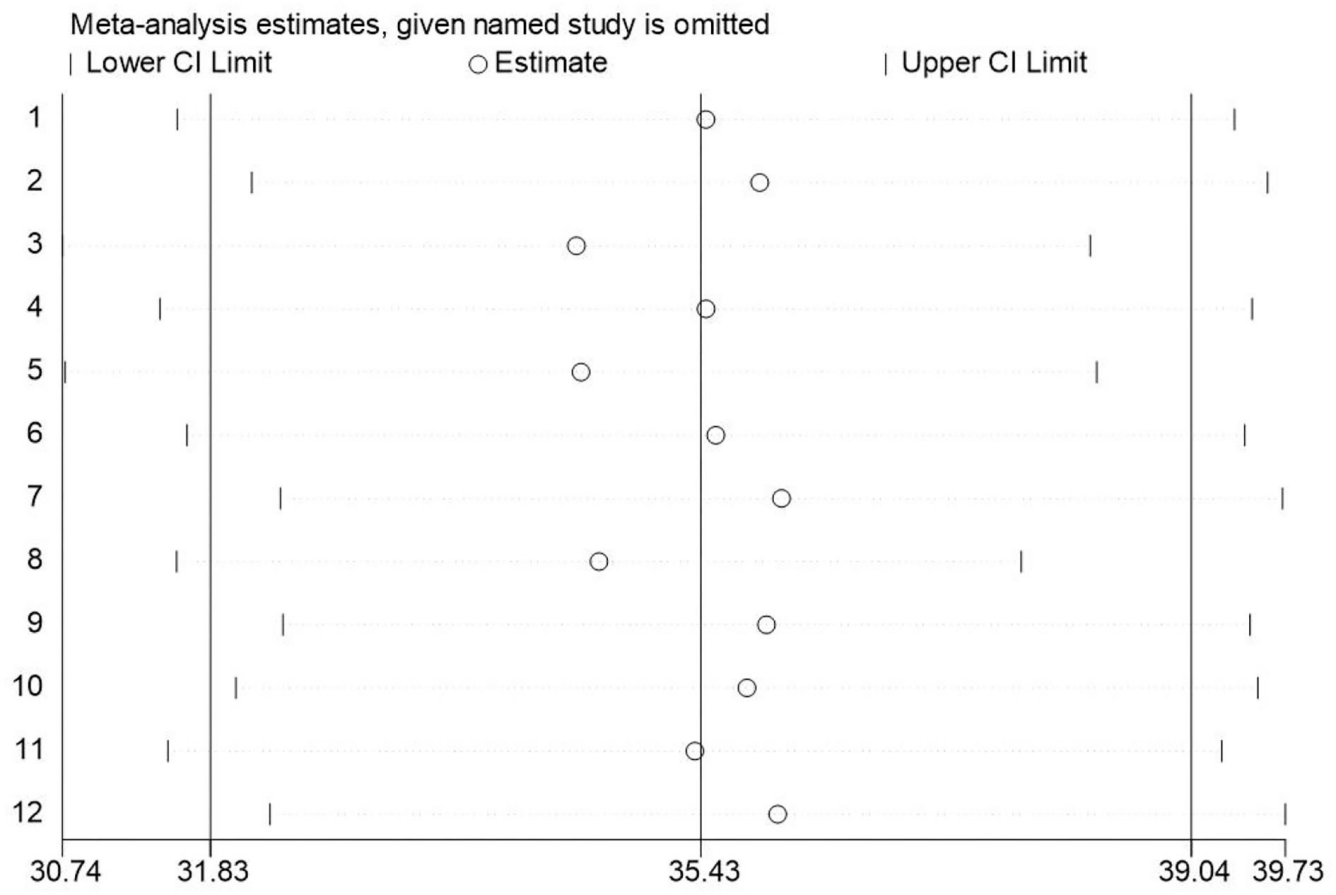
Sensitivity analysis.

A sensitivity analysis was performed, and the results were not significantly different from the total combined estimated value, which suggested that the meta-analysis results were relatively stable and reliable (Figure 3). Begg's test was used to evaluate publication bias, and a score of (*Z* = 0.945) suggested that the risk of publication bias in this study was low.

## Discussion

The Newcastle-Ottawa scale (NOS) score ranged from 6 to 8 points, indicating that the quality of the included studies was high. The results of the meta-analysis showed that the score of Chinese nurses' work alienation was 35.43 [95%*CI* (31.88, 39.04)], which was at a moderate level. However, this score was higher than Turkish nurses (15) and Dutch midwives (9), but lower than Pakistani middle managers (5) and Iranian workers (34). The reasons for this difference may be related to occupation, national policies, and varying medical and health service systems.

Male nurses had a higher level of work alienation than female nurses, and the result was consistent with the finding of Hu (23). Male nurses are under greater work pressure due to their lower social acceptance in China. They have a poorer sense of group belonging, lower professional identity, and a stronger willingness to resign, so the level of work alienation is relatively higher among men (35, 36). Therefore, managers should assign male nurses responsibilities that are in accordance with their logical thinking ability and calmness in case of trouble so as to maximize their potential; on the other hand, professional training for male nurses in the department should be implemented regularly, and timely encouragement and support should be given to increase their professional identity and sense of group belonging, thereby reducing male nurses' sense of work alienation.

The results of this study showed that with higher salaries, nurses' work alienation score gradually decreases, which was consistent with the findings of Tummers (9). Half of the nurses in China are dissatisfied with their income, which, to some extent, reflects the inability to fully fulfill the professional value of nurses (37). Nurses with higher incomes have higher job satisfaction and professional identity. They can be intrinsically motivated in the process of career development and have the enthusiasm to actively participate in the work, so the level of work alienation is lower (38). Flodgren et al. (39) believed that the compensation incentives for medical staff were conducive to motivating medical staff to change their behaviors in decision-making, improve patient prognosis, and thus improve the quality of care. Therefore, hospitals should appropriately increase nurses' remuneration and benefits, establish reasonable internal and external incentive mechanisms, and reduce the level of nurses' work alienation, which is of great significance to stabilizing the nursing team, promoting their work enthusiasm (40), and reducing the turnover rate of nurses.

Nurses with higher education levels had higher levels of work alienation, which was inconsistent with the finding of Wei (41). Wei pointed out that people with high education levels establish flexible work arrangements at work because of the advantages brought by their academic qualifications, abilities, and learning patterns, and they can arrange work more flexibly and have greater autonomy in work (41). According to our study, nurses with higher education levels are usually engaged in clinical nursing as well as scientific research work. Such overloaded work reduces their professional identity and job satisfaction (42), which results in a sense of work alienation. Therefore, department managers should formulate training programs for nurses to choose either specialization in scientific research or clinical nursing. Nurses with higher education levels should focus on training as scientific research nurses while reducing clinical nursing responsibilities, and specialist nurses should focus on consolidating specialist nursing knowledge and improving their professional skills.

The results of this study showed that with the promotion of professional titles and the increase of clinical working time, the level of nurses' work alienation gradually decreased, which was inconsistent with the findings of Sulu S, Kocoglu (43, 44). Nurses with lower levels of professional titles and shorter clinical working time may lack work experience and interpersonal skills, and the probability of erroring in clinical nursing is higher than that of nurses with higher professional titles, which then leads to job dissatisfaction and work inefficiency, hence a higher level of work alienation (45). Therefore, for nurses with lower professional titles and shorter clinical working time, managers should rationalize their shifts, reduce work intensity, and gradually guide nurses to adapt to higher-intensity workloads, thereby reducing clinical nursing errors. At the same time, managers should focus on nurses' psychological state and take measures such as structural empowerment and strengthening peer mutual assistance to improve their psychological resilience. This would improve their wellbeing and professional identity (46), thereby alleviating their work alienation and finally reducing the nurse turnover rate.

According to our study, unmarried nurses have higher work alienation scores than married nurses, which was consistent with the finding of Ren (19). Married nurses tend to rely on family and social support when they feel alienated at work. However, unmarried nurses cannot constantly obtain family support since they have been independent of their parents while still in need of spouses. Sometimes they cannot effectively resolve traumatic events at work and negative emotions may persist, finally amounting to work alienation (41). Therefore, nursing managers should provide unmarried nurses with emotional support and care. At the same time, nursing managers are obliged to improve the social support system and create good interpersonal relationships to improve the overall quality of nurses' work and life (47).

This study shows that nurses in internal medicine departments, emergency departments, and intensive care units have higher scores of work alienation. While the results of Gao et al. (48) showed that pediatric nurses had higher work alienation than internal medicine nurses, which may be a result of the small sample size—only 31 pediatric nurses were included.

With the current increase in the older adult population, China is facing challenges and difficulties in terms of the medical demand for chronic diseases. Most of the patients in the internal medicine department are frail and senile, suffering from complex illnesses. So, a higher quality of professional care and interpersonal communication skills are required, which, however, proves to be a source of great pressure for nurses. Once the pressure is too high and they cannot relieve it, it is easy to feel alienated at work. The high score of work alienation of nurses in the emergency departments and the intensive care units is consistent with the research results of Wu et al. (49). Compared with other departments, the work in the emergency departments and the intensive care units is of a faster pace, higher load, and higher risk Nurses in these departments and units tend to have a negative and exhausted mentality, and work alienation occur frequently. Managers should make reasonable human resource arrangements according to the situation of the department, conduct regular psychological counseling for nurses, and organize relevant team-building activities to improve cohesion among the nurses. At the same time, managers should reasonably allocate the workload of nurses, with human resources scientifically equipped to reduce their workload (50), thereby reducing nurses' sense of work alienation.

Chinese nurses have a moderate level of work alienation. However, males, higher education, lower professional titles, unmarried, shorter working time, lower income, internal medicine, and critical care nurses have higher work alienation scores. So, early identification of work alienation and timely intervention are needed to reduce the turnover rate of nurses and improve the quality of nursing. This study has certain limitations: (1) this study was uncontrolled binary data, and the inclusion of the literature was more extensive, which increased the heterogeneity between the studies; (2) during the meta-analysis, studies that did not use the scale compiled by Ren or were unable to calculate the score from the literature were excluded, which may impact the final combined effect size; (3) all the studies were conducted in eastern and central China. Larger samples, high-quality, and multi-center original research will need to be conducted in the future.

## Implications and future research directions

This study has demonstrated the status of work alienation among nurses in China. Learning the status of work alienation can provide an up-to-date theoretical basis for the management of nurses. Most significantly, this study raises the awareness of work alienation among healthcare managers.

Three possible future research directions can be identified from our study. First, at present, there exist only a very small number of studies on work alienation among nurses, compared with studies on work alienation among other professionals, and few focused on the predictors or the consequences of work alienation. Future research should use experimental design or longitudinal designs for strong causal insights (51). Second, work alienation is a subjective negative psychological state; therefore, qualitative and quantitative studies should be conducted to explore the understanding and experiences of work alienation among nurses so as to gain in-depth knowledge about which interventions are effective to reduce this feeling, and so on. Third, work alienation has been proven to be a crucial mediator and/or moderator for the relationship between organizations and employees. For example, a previous study indicated that work alienation was an important mechanism through which laissez-faire leadership influenced job burnout (52). Therefore, similar studies should be carried out to explore the potential links between organizations and employees and extend the existing literature.

## Author contributions

Conception and design: QY, JG, and CH. Administrative support: DB, CW, and CH. Provision of study materials or patients, data analysis, and interpretation: QY, DB, and CW. Collection and assembly of data, manuscript writing, and final approval of manuscript: all authors.

## Funding

The study was supported by Sichuan Nursing Association (grant no: H20003).

## Conflict of interest

The authors declare that the research was conducted in the absence of any commercial or financial relationships that could be construed as a potential conflict of interest.

## Publisher's note

All claims expressed in this article are solely those of the authors and do not necessarily represent those of their affiliated organizations, or those of the publisher, the editors and the reviewers. Any product that may be evaluated in this article, or claim that may be made by its manufacturer, is not guaranteed or endorsed by the publisher.
